# Barriers and Determinants to the Underutilized Hypertension Screening in Primary Care Patients in Hong Kong: A Mixed-Method Study

**DOI:** 10.3390/ijerph20020985

**Published:** 2023-01-05

**Authors:** Rachel Yui-Ki Chu, Dong Dong, Samuel Yeung-Shan Wong, Eric Kam-Pui Lee

**Affiliations:** 1 The Jockey Club School of Public Health and Primary Care, Faculty of Medicine, The Chinese University of Hong Kong, Hong Kong SAR, China; 2 Shenzhen Research Institute, The Chinese University of Hong Kong, Shenzhen 518000, China

**Keywords:** hypertension, primary healthcare, screening, blood pressure, mixed method

## Abstract

(1) Background: Hypertension (HT) is the most common chronic condition, affecting approximately 1.13 billion people worldwide. Despite freely available blood pressure (BP) devices in primary care (PC) clinics, many patients do not regularly screen for HT and are untreated. (2) Methods: This study investigated the proportion of PC patients who did not screen for HT and the underlying reasons in Hong Kong. An explanatory mixed-method cross-sectional study was conducted in 2020, which included a questionnaire survey, office BP measurements, and subsequent semi-structured interviews. Adult patients who had no diagnosis of HT were recruited in a large PC clinic by convenience sampling. The relationships between not having HT screening and sociodemographic data were investigated by logistic regression. Twenty-four patients were purposefully sampled (based on demographics) and were interviewed until data saturation. (3) Results: Among 428 participants, 190 (44.4%) had not had HT screening in the last two years, but 197 (46.0%) had HT. No HT screening in the last two years or ever was associated with being male, being single, being of younger age, having no family history of HT, having no clinic visits in the last two years, employment status, and self-perceived HT condition. Most participants (77.8%) misinterpreted their BP readings. Individual, social, and healthcare service barriers were identified in patients’ interviews. Many PC patients had no regular HT screening but around half had elevated BP. (4) Conclusion: The study results indicate that the barriers to HT screening were multifactorial. HT screening in PC is urgently needed.

## 1. Introduction

Hypertension (HT) is the most common chronic condition, affecting around one-third of the adult population worldwide [1]. Approximately 1.13 billion people are suffering from HT worldwide [2]. HT is the major cause of cardiovascular mortality, accounting for 9.4 million deaths annually [2,3]. Due to the asymptomatic nature of HT, international guidelines and the Hong Kong (HK) guideline recommend regular HT screening, which is cost-effective for preventing cardiovascular complications through early and effective treatments [4,5].

Despite these recommendations, regular screening of HT is often not conducted. Around 30–50% of patients with HT are unaware of the condition and are, therefore, untreated [1,6]. A systematic review identified patients’ barriers to HT awareness, which included: knowledge, skills, motivation, social influences on HT screening, availability of screening, and other social determinants [7]. These barriers were identified in both developing and developed countries, the elderly, and multi-ethnic populations [8]. Around half of the people with elevated blood pressure (BP) in HK were unaware of their condition in a population-based survey [4]. 

Although the prevalence of people who did not screen for HT in the general population and their corresponding barriers were well described, similar research was yet conducted in primary care (PC). Screening in PC may be more cost-effective by targeting high-risk patients because HT is associated with numerous other diseases [8,9]. Although an official screening program for HT is lacking in HK, regular HT screening is promoted in doctors’ offices and hospitals. BP devices are provided freely in social centers and publicly funded general outpatient clinics (GOPCs). Once diagnosed, HT treatment is readily available and affordable in GOPCs. In contrast to the general population, we hypothesize most PC patients received HT screening due to convenience and free service in GOPCs, and the reasons for not having HT screening would be different.

This is a sequential explanatory mixed-method study. For primary aim, the proportions of patients who have not been screened for HT in the last two years or ever were estimated. For secondary aims:

(i) patients’ demographic factors associated with no HT screening were identified.
(ii) Barriers to HT screening were identified by qualitative interviews. Patients who had not had HT screening in the past two years, as identified from the survey, were purposefully sampled based on their demographics, which is expected to further explain findings from (i).

The results would facilitate researchers, clinicians, and policymakers to promote HT screening in primary care, which may thereby reduce cardiovascular events and deaths.

## 2. Materials and Methods

This study was approved by the Joint CUHK-NTEC Clinical Research Ethics Committee (CREC Ref. No.: 2021.076). Written consent was obtained from all participants.

### 2.1. Participants

Participants (aged ≥ 18) were recruited from a large GOPC (Lek Yuen clinic) in HK from April to June 2020. Participants who fulfilled any of the following criteria were excluded: (i) diagnosed with HT, diabetes mellitus, stroke, heart diseases, or chronic kidney diseases because BP measurements were compulsory for these patients, (ii) unable to communicate in Cantonese, Mandarin, or English, (iii) had a physical or mental illness that prevented the BP measurements, or (iv) unable to consent.

### 2.2. Instruments

As there is no well-validated questionnaire to detect the proportion of patients with no HT screening, a questionnaire was designed by a team of academics including 2 family medicine specialists and academics and 1 public health academic after reviewing the existing literature. The questionnaire was designed to collect (i) demographic data (e.g., age, sex, education level, presence of a regular family doctor), (ii) whether the patient had ever conducted HT screening, and in the last two years, and (iii) whether they interpreted their BP correctly (see BP measurements). The questionnaire was piloted in 20 primary care patients prior to data collection to ensure relevancy and clarity. The questionnaire can be found in (Supplementary File S1). 

### 2.3. BP measurements and Body Mass Index

These are collected because no HT screening may associate with elevated BP because of under-diagnosis. Furthermore, obesity is a known risk factor for HT and may prompt regular HT screening. 

After the questionnaire survey, BP was measured by a trained master student using an office BP monitor TM-2657P (A&D Company, Limited, Tokyo, Japan), which was validated according to the British and Irish Hypertension Society standards. BP was first measured on each arm. Two further BP readings were obtained for analysis and averaged using the arm with higher BP. Elevated office BP was defined as systolic BP of ≥140 mmHg or diastolic BP ≥ 90 mmHg [10]. Body weight and height were measured using the validated MUW 300 L ultrasonic Health and fitness scale (Adam Equipment Company, Milton, UK).

### 2.4. Sample Size Calculation for Questionnaire Survey

Using a margin of error of 5%, a confidence level of 95%, and the presumed proportion of patients who had not received screening for HT to be 50% (which required the largest sample size), 384 participants were needed. To allow a dropout rate of 10%, at least 426 participants were required.

$$n=\frac{z^{2}\times \hat{p}\left(1-\hat{p}\right)}{\epsilon^{2}}$$

$$n=\frac{1.96^{2}\times 0.5\left(1-0.5\right)}{0.05^{2}}=384.16$$

*z* is the z score.
*ϵ* is the margin of error.
*n* is the population size.
$\hat{p}$ is the population proportion.

### 2.5. Qualitative Interview 

The inclusion criteria of the qualitative interview were patients (1) who had not screened for HT in the previous two years; and (2) who had been found to have elevated BP during the BP measurements as part of the quantitative survey. Patients meeting the inclusion criteria were further sampled based on age, gender, marital status, employment status, clinical visit in the past two years, and self-perceived HT condition. 

A semi-structured interview guide was developed based on the questionnaire results and literature review (Supplementary File S2). Interviews were conducted by the trained master student via telephone due to the COVID-19 pandemic. Each interview lasted from 15 to 30 min. Preliminary qualitative data analysis was conducted immediately after each interview. Interviews were continued until data saturation (no new information can be further identified) [11].

### 2.6. Statistical Analysis 

Quantitative data was performed using the Statistical Package for Social Science version 25.0 (SPSS, IBM Corp., Armonk, NY, USA). Demographic data were presented as mean with standard deviation and numbers with percentages for continuous and categorical data, respectively. Univariate analysis and Mann-Whitney U test were performed to examine the association between no HT screening and sociodemographic data. Significant factors from the univariate analysis were further analyzed by simple logistic regression. Statistical significance was defined as *p*-value < 0.05. Interviews were audio-recorded and transcribed verbatim. Two investigators (RYKC, DD) repeatedly read and familiarized themselves with the transcripts. Thematic analysis was conducted, in which concepts were coded, and grouped into patterns/themes using excel (Microsoft 365 Excel Version 2021) [12]. Quantitative and qualitative results were compared and presented in a side-by-side table.

## 3. Results

### 3.1. Quantitative Results

#### 3.1.1. Participant Characteristics

**Figure 1:**
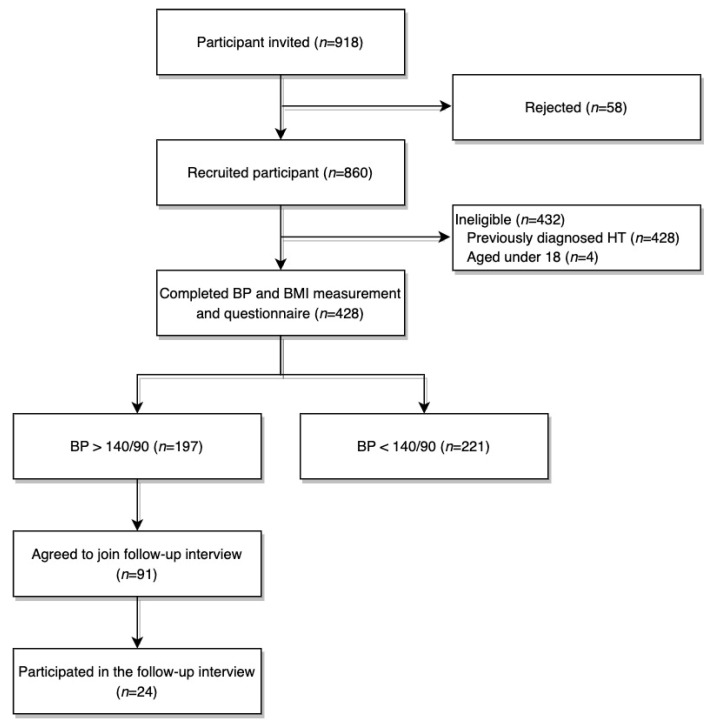
Number of participants at each stage of the study.

Among 918 patients who were approached, 432 were excluded, mostly due to diagnosed HT. (428 were known HT patients, and 4 were aged under 18). A total of 428 participants were included (Figure 1). 

The mean age and BMI of the participants were 56.2 ± 15.6 years and 24.2 ± 3.7 kg/m^2,^ respectively. Most participants were female (50.7%), were employed (46.7%), received at least secondary education (70.1%), were married (80.1%), were without self-reported chronic diseases (71.0%), were never smokers (82.2%), were never drinkers (71.5%) and were without health insurance (70.8%). Most did not have a regular family doctor (87.9%) but had visited a doctor in the previous two years (71.7%) (Table 1).

#### 3.1.2. The Proportion with no HT Screening in GOPC

In total, 146 patients (34.1%; 95% confidence interval [CI]: 29.6–38.8%) and 190 patients (44.4%; 95%CI: 39.6–49.2%) had never had BP measurements and had not had BP measurements in the previous two years, respectively (Table 2). Furthermore, 197 patients (46.0%; 95%CI: 41.2–50.8%) had elevated BP, while 333 (77.8%; 95%CI: 73.6–81.7%) participants misinterpreted their BP results.

#### 3.1.3. Factors Associated with no HT Screening in the Previous Two Years or Ever

Similar factors were associated with no HT screening in the past two years and never having had HT screening. In univariate analysis, these factors included gender, marital status, employment status, family history of HT, having a clinic visit in the last two years, and age. Regarding their BP values as normal was associated with no BP measurements in the last two years only. In logistic regression, no HT screening in the last two years or ever was associated with being male, being single, being of younger age, having no family history of HT, and having clinic visits in the last two years. Patients who were not able to interpret their BP values had higher odds of not having had HT screening in the last two years (Table 2).

### 3.2. Qualitative Results

Patients who participated in the survey and found themselves to have HT only through this study were interviewed via telephone. They were sampled based on their age, gender, marital status, employment status, and clinical visit in two years, and they considered their BP as normal. Interviews continued until data saturation was reached (Supplementary Table S1 shows details of interviewees). Eventually 24 interviews were completed. Since the purpose of the follow-up qualitative interviews was to identify the barriers for these patients not to have BP measurement, three overarching themes concerning their barriers were identified: (1) Individual barriers; (2) Social barriers, and (3) Healthcare service barriers (Table 3).

Barriers to individual patients not having HT screening vary; yet they can broadly be summarized at three levels: individual, social, and institutional. The barriers at the individual level can be further divided into two sub-themes: (1) insufficient knowledge about HT and (2) feeling suspicious, careless, or even in denial of HT. 

#### 3.2.1. Individual Barriers: Insufficient Knowledge about HT

We were surprised to find out that, regardless of their age or gender, many patients had many misconceptions about HT. For example, the majority of participants expressed their HT knowledge insufficiency. When they were asked about the BP index classification, most of them did not know or failed to answer correctly. A number of participants reported that a BP over 140 is normal (participant 5, 7, 20, and 22). Some explained they had experienced difficulties in reading the index and were unable to interpret it which become a major barrier in measuring BP. 

In addition to BP index interpretation, the knowledge of HT’s nature is unclear regardless of age. Some patients thought that elevated BP was just a temporary thing, and it would not cause any harm to their health. An 18-year-old and a 50-year-old participant both believed HT is temporary and that BP will return to normal shortly (participant 16 and 17). Over half of the participants perceived they were in good health and that they never expected to have HT. A middle-aged female participant further explained that it is unexpected to have HT as she is still young, and her body is in a good shape (participant 4). Such misconceptions might also happen to those who perceive themselves as having quite high health literacy. A 62-year-old man who claimed to read medical books stated that HT and diabetes could be felt by oneself (Participant 7), but, apparently, his knowledge about HT was also off base. With the asymptomatic nature of HT, it could be difficult for patients to realize that they were sick by themselves. In comparison, patients who admitted having low health literacy also felt relaxed about being found to have HT, but the main reason was that they did not know the meanings of the BP numbers (participant 3, 4, and 10). 

#### 3.2.2. Individual Barriers: Feeling Suspicious, Careless, or Even Denial 

At the attitudinal level, men or women, most patients were optimistic about their current and future health. Two interviewees commented that they were healthy without any health problems and living normally. They further emphasized that measuring BP is unnecessary for them as they will never get HT (Participant 4, 6, and 22). After our BP measurements, some interviewees denied having HT and claimed that “medical consultation is meaningless” (Participant 19). A 63-year-old female later explained that the reason why she refused to measure BP was that she understood her lifestyle was unhealthy and was reluctant to face that reality (Participant 12). 

The denial attitude was further demonstrated through their behaviors. Some patients showed distrust towards office BP devices and believed that measuring BP was a waste of time; some suspected the BP machines were broken, especially when they were found to have a high BP (Participant 24). One even accused the measurements of being high and inaccurate for everyone (Participant 19). 

#### 3.2.3. Social Barriers

At the social level, work constraints and insufficient social support were the most salient social barriers that emerged from the interviews. The working patients could not optimally take care of their health due to insufficient time and occupation by their work schedule. This phenomenon appeared in employed young to middle adults. A 24-year-old employed young adult reported that the packed work schedule disrupted his daily health routine. He further supplemented that he had insufficient time to sleep, and, thus, it was barely possible for him to have time to measure his BP (Participant 2). Participant 2 also mentioned that his working environment prohibited him from adopting a healthy lifestyle including doing physical activities and choosing healthy food. Another working adult (Participant 19) complained that he had no spare time to consider healthy food choices as he was fully occupied by work. A similar situation was experienced by Participant 4 as she expressed that there were limited healthy food choices around her workplace. 

Lacking social resources and support could preclude HT screening. In particular, the elderly lacked medical knowledge, and skills, thereby requiring extra support for HT screening. They had difficulties in interpreting BP values and required assistance with other healthcare needs. An 80-year-old lady expressed her concern about measuring BP because she was not able to take care of her health when her children were out for work, plus there was no extra social support provided by the government to assist her after she left the clinic (Participant 3). Another 83-year-old man commented that district counsellors would measure BP for them in the past, but there had not been such services for a long time (Participant 20). 

#### 3.2.4. Healthcare Service Barriers

At the institutional level, despite free and available BP devices in GOPCs, patients often perceived a shortage and reduced accessibility of BP machines in the communities. A participant reported the decreased number of BP devices in the GOPC and difficulty finding BP devices in his own community (Participant 22). With the limited availability of BP devices, it became more difficult for people to monitor or screen their HT. In addition, some interviewees who lived in poverty disclosed their hardship to afford a BP device at home. The decreased number of public BP devices indirectly discouraged their BP measurement (Participant 14). 

### 3.3. Mixed-Method Analysis Results

To juxtapose and compare the findings from quantitative surveys and qualitative interviews, further insights on the reasons for patients not having HT screening emerge. Simply put, qualitative results echo and provide further evidence to support our quantitative findings. The comparison of quantitative and qualitative findings is listed in Table 4.

In our quantitative findings, numbers of factors were found to have an association with no HT screening in the past two years: male, single, younger age, unemployed, without a family history of HT, and without clinic visits in the last two years. Aligning these findings with the qualitative results, our further explanation on such associations are: (1) the younger age was associated with no HT screening as they regard HT will only affect older people; (2) being single means that no family or spouse would have experienced BP-related diseases, paid more attention to their BP, or helped them maintain their health; (3) for individuals without a family history, they would consider themselves as low-risk population; and (4) absence of clinic visit in 2 years, and in combination with the insufficient BP devices provided in the community, both demotivated individuals in BP measurement. The qualitative results, however, cannot further explain the gender differences.

In addition, from the survey, some patients were found to be unable to correctly interpret their BP values, which can be further explained by poor HT knowledge among the patients who might also consider HT screening as irrelevant, as the qualitative results show. Subsequently, these patients had a wrong belief in their BP conditions and, therefore, avoided HT screening. 

## 4. Discussions

### 4.1. Summary

This was the first mixed-method study in PC exploring the prevalence and reasons for no HT screening. Around 44% of PC patients had not received HT screening in the previous two years despite free screening. Population-based studies indicated around one-third of the Chinese population were unaware of their HT [6]. Aligning with a systematic review, knowledge, attitude, and social factors played important roles in not receiving HT screening; however, this was the first study to elicit prevalence and barriers specific to PC patients [13,14]. Our study suggests that regular universal HT screening in PC would identify a large number of undiagnosed patients with HT because approximately one-third of our participants had elevated BP whilst 80% were unable to interpret their BP values. 

### 4.2. Implication for Practice

Our results suggest that despite free HT screening in the PC setting, many patients did not receive HT screening due to various reasons. At individual levels, patients should be educated about the details of screening (including the threshold of HT diagnosis, and proper BP measurement techniques), complications, prognosis, and treatments of HT. It is commonly found that there is a lack of medical knowledge among the elderly, and extra social support is encouraged [15]. Our result shows that those of younger age perceived themselves as a low-risk population whereas further public health education should be provided to clear the misconception. A denial attitude is typical when patients are first told that they have some unexpected disease [16]; a deeper medical knowledge may assist patients with disease acceptance. At clinical practice levels, if universal screening for all patients during every clinical visit is infeasible, computerized systems can be designed to alert healthcare professionals when the patient has had no screening in the previous two years. The threshold for HT diagnosis should be posted in the clinics. Free BP measurements can be made available in clinics and in the community to facilitate regular screening [17]. Finally, governments can set up universal screening programs and reimburse clinicians to make definite HT diagnoses by out-of-office BP methods, which have superior reproducibility and predictability to cardiovascular events than office BP measurements. Out-of-office BP is recommended for HT diagnosis [18,19]. Our results provided important patient characteristics and their reasons for not receiving HT screening which can guide HT screening strategies.

### 4.3. Implications for Research

Our results showed that implementation studies integrating evidence-based HT screening, which is shown to reduce hospitalizations and deaths, into PC are urgently needed [20]. This is hardly unique to HT screening because it took 17 years on average for evidence-based interventions to be successfully implemented into daily practice [21]. Our results suggest that employed participants had difficulties receiving HT screening, and it is currently unclear whether implementing BP devices in the workplace is feasible, accurate, or can improve HT detection. Although clinic BP was recommended to be the yearly screening tool for HT in all adults, it only has fair accuracy to diagnose HT (sensitivity of 0.54 [95% CI, 0.37–0.70] and specificity of 0.90 [95% CI, 0.84–0.95]) [10,22]. More studies may determine whether home BP measurement that is more sensitive, specific to diagnose HT than office BP, and well-accepted by HT patients can be a valid and accepted regular screening tool for HT in PC patients [23,24].

### 4.4. Strength and Limitations

The current mixed-method study was one of the first to explore the proportion and the patients’ reasons for not receiving HT screening. The sample size was pre-determined and adequate. The qualitative interview methodology was guided by, subsequently explained, and provided a deep understanding of the quantitative results.

A few limitations should be discussed. Although recruitment was carried out in a single large publicly funded center, the applicability of our results to other clinics, especially private clinics, was unknown. In HK, relatively wealthy and educated patients may prefer private general practitioners. However, >80% of patients with chronic diseases received care from public PC clinics [19]. As a cross-sectional study, causal relationships cannot be established. Although we found that patients without HT screening did not have an increased risk of elevated BP, patients at risk of having elevated BP may measure more frequently and were excluded from our study once they were diagnosed with HT. BP was measured only in one clinic visit. To definitely diagnose HT, BP measurements over a few clinic visits or using out-of-office BP measurements are recommended, but these were infeasible for this entirely self-funded study [10]. 

## 5. Conclusions

Around 44% of PC patients had no regular HT screening and many unscreened patients had elevated BP. Barriers to HT screening were multifactorial and consisted of individual, social, and healthcare factors. Gender, marital status, employment status, family history, clinic visits in two years, and age were associated with no BP screening from the quantitative result, whereas qualitative results supported the identified three barriers that hindered individuals in measuring BP. The individual barrier included knowledge and attitude towards HT; the social barrier included work constraints and lack of care; and the health service barrier reflected the insufficiency of resources in the community. PC doctors should encourage regular screening for HT. On top of that, health education is necessary to clear the misconception of HT and deepen public knowledge of BP. Extra care is particularly needed for the elderly in BP management. Studies investigating the implementation of HT screening in PC are urgently needed.

## Figures and Tables

**Table 1:** Participants Characteristics.

Independent Variables	*n*	%	Mean (SD)
All participants	428		
Gender			
Female	217	50.70%	
Age			56.2 (15.6)
Marital status			
Not married	85	19.90%	
Married	343	80.10%	
Measured level of BP			
BP ≥ 140/90	197		
BP < 140/90	231		
Self-perceived BP condition			
Normal	120	28.00%	
Abnormal	36	8.40%	
Do not understand BP index	272	63.60%	
Family history of HT			
Yes	187	43.70%	
No	204	47.70%	
Don’t know	37	8.60%	
Clinic visit in 2 years			
Yes	307	71.10%	
BMI			24.7 (9.6)

BMI = body mass index, BP = blood pressure, HT = hypertension.

**Table 2:** Association between clinical/demographic data and not receiving HT screening.

Independent Variables	Never Measured BP (% within Groups)	Univariate	Regression		Not Measure BP in 2 Years (% within Groups)	Univariate	Regression	
		*p*-Value ^†^	*p*-Value *	Odd Ratio		*p*-Value ^†^	*p*-Value *	Odd Ratio
Gender		0.004	0.004			0.007	0.007	
Female **	59 (31.2%)				81 (39.1%)			
Male	87 (45.5%)			1.843	109 (52.4%)			1.713
Age		<0.0001 ^‡^				<0.0001 ^‡^		
Marital status		<0.0001	<0.0001			<0.0001	<0.0001	
Not married **	44(55.7%)				52 (65.80%)			
Married	102 (29.2%)			0.341	138 (41.1%)			0.362
BP measurement		0.001				0.003		
BP ≥ 140/90	49 (28.8%)				71 (37.8%)			
BP < 140/90	97 (46.2%)				119 (52.4)			
Self-perceived BP condition		0.019	0.017			0.005	0.004	
Normal	35 (31.8%)			1.66	45 (38.5%)			0.588
Abnormal	8 (23.5%)			2.417	10 (27.8%)			0.362
Do not understand BP index **	103 (43.6%)				135 (51.5%)			
Employment status		0.003	0.003			0.005	0.004	
Employed	75 (41.7%)			1.597	95 (48.5%)			1.411
Unemployed	16 (51.6%)			2.384	19 (57.6%)			2.036
Retired	30 (28.8%)			0.906	43 (37.1%)			0.884
Student	8 (80.0%)			8.941	9 (90.0%)			13.5
Housewife **	17 (30.9%)				24 (40.0%)			
Marital status		<0.0001	<0.0001			<0.0001	<0.0001	
Not married **	44(55.7%)				52 (65.80%)			
Married	102 (29.2%)			0.341	138 (41.1%)			0.362
Family history		<0.0001	<0.0001			<0.0001	<0.0001	
Yes	47 (27.8%)			0.317	63 (34.8%)			0.365
No	82 (45.6%)			0.689	108 (53.5%)			0.786
Don’t know **	17 (54.8%)				19 (59.4%)			
Clinic visit in 2 years		0.001	0.001			0.001	0.001	
Yes	92 (33.5%)			0.475	122 (40.7%)			0.474
No **	54 (51.4%)				68 (59.1%)			
BMI		0.757				0.527		

^†^ Chi-square test, ^‡^ Independent *t*-test, * Binary logistic regression, ** Reference group, BP = blood pressure, HT = Hypertension, BMI = Body Mass Index.

**Table 3:** Overview of the themes, sub-themes, codes and quotes.

Themes	Sub-Theme	Codes	Quotes
Individual barrier	Insufficient knowledge about HT	Low perceived severity	“I was not aware of the information about hypertension until today when I was told to have hypertension. I know it is difficult to manage.”—participant 13, male, aged 74
			“I don’t know if it is high or not, and it won’t cause death. I am not fat and still young, I didn’t expect I would have high blood pressure.” Participant 4, female, aged 53
			“I didn’t know HT would cause harm to my body.”—participant 5, male aged 43
		Wrong interpretation of HT	“My Blood pressure is not high; it’s not over 150 yet.” participant 5, male, aged 43
			“My BP is 140 which is in a normal range.” Participant 8, male, aged 62, Participant 21, male, aged 83 & Participant 23, male, aged 72
			“I am not scared as I don’t understand what the number means. Why would I measure if I don’t know what it means?”—participant 3, female, aged 80; participant 4, female, aged 53 & participant 10, female, aged 72
		Thought it will recover over time	“I think the blood pressure will return to normal afterwards.”—participant 16, female, aged 18 & participant 17, male, aged 50
		HT would not lead to death	“I don’t know if it is high or not, and it won’t cause death. I am not fat and still young, I didn’t expect I would have high blood pressure.”—participant 4, female, aged 53
		HT does not cause harm to health	“That was just a number to me, I don’t even know if it is high or low. I don’t know what I can do after the measurement. What is the point of measuring? It is so disturbing!”—participant 6, male, aged 19
		Misunderstanding on the pathophysiology of HT	“I would know if I had hypertension. I can feel it if I have it, even diabetes can be felt by yourself. I also read medical book, so I know I don’t have hypertension.”—participant 7, male, aged 62
		Low health literacy in general	“I am not scared as I don’t understand what the number means.”—participant 3, female, aged 80; participant 4, female, aged 53 & participant 10, female, aged 72
	Feeling suspicious, careless or even denial	Low self-awareness	“I’m too young to get into this (HT)”—participant 16, male, aged 22 and participant 6, male, aged 19
		Low sense of urgency	“Why do I need to measure (blood pressure) at a young age?”—participant 16, female, aged 18
			“My family does not have a history of hypertension; I don’t think I need to be worried.”—participant 23, male, aged 22
		Denial	“I did not pay attention, so I did not measure. I don’t care at all; medical consultation is meaningless.”—participant 3, female, aged 80
		Optimistic about their own health	“I think I am healthy without any severe diseases, and I am able to walk and eat normally. I don’t think I will have hypertension.”—participant 4, female, aged 53, participant 6, male, aged 19 & participant 21, male, aged 81
			“My family and spouse got hypertension as well; I think I don’t have hypertension.”—participant 7, male, aged 62 & participant 4, female, aged 53
		Suspicious of the BP machine accuracy	“I think the machines at the clinics are broken.”—participant 17, male, aged 50
Social barrier	Work Constraints	Work disrupting healthy lifestyle	“I have no time for sleep; how would I have time to take care of my health, say measure BP?”—participant 2, male, aged 24
		Lack of physical activity	“Working inhibits me from doing physical activities. There is a limited choice of food in my canteen. I would work out and eat healthily before joining the company”—participant 2, male, aged 24
		Unhealthy eating habits	“I did not pay attention to choosing healthy food as there is not much promotion of healthy food around my workplace. Sometimes my emotions quite fluctuate which might also lead to hypertension?”—participant 4, female, aged 53 & participant 2, male, aged 24
			“I am too busy at work and do not wish to spare time on healthy food choice.”- participant 18, male, aged 31
	Lack of informal care	Lack of care from social support	“I cannot take care of my own health. No one takes care of me as they all go to work. The government did not offer assistance when I left the clinic”—participant 3, female, aged 80
			“I haven’t measured it for a long time. The district counsellor measured BP for us, but there are no such events now.”—participant 20, male, aged 83
Healthcare service barrier	Insufficient resources in the society	Hard to find BP machine	“The number of BP machines in the GOPC reduced from 7 to 3. It is not easy to find one in the community.”—participant 22, male, aged 72
		Financial difficulties	“If I have to measure, I have to go somewhere to find the BP measuring machine, else I would have to spend several hundred to buy one!”—participant 14, male, aged 75
		BP machine too expensive for individuals with low SES	

**Table 4:** Summary of the mixed-method result.

Quantitative Findings	Qualitative Findings
Although HT screening in GOPC is free and encouraged, most patients do not screen for HT	Multiple individual, social and healthcare barriers were identified
Most patients misinterpreted their BP values	Patients had poor knowledge about HT
Younger age was associated with no HT screening	Younger patients regard HT as a disease only affecting elderly people
Being a student or unemployed were associated with no HT screening	Low availability in living surroundings and schedule constrictions hindered BP measurement
Being single was associated with no HT screening	Patients’ spouses might have experienced BP related diseases which would lead to a higher awareness; No caregivers at home to provide assistance
Having no family history of HT was associated with no HT screening	Low awareness on measuring BP was found among patients without a family history of HT as they considered themselves to be a lower risk population
Absence of clinic visit in 2 years was associated with no HT screening	A lack of BP devices in the community hindered HT screening
Patients who could not interpret their BP values and those who regarded their index as normal tended not to measure	Patients regarded their BP as normal and considered themselves as healthy

## Data Availability

The data used and analyzed during the current study are available from the corresponding author upon reasonable request.

## Supplementary Materials

The following supporting information can be downloaded at: https://www.mdpi.com/article/10.3390/ijerph20020985/s1, File S1: Questionnaire; File S2: Interview guide; Table S1: Characteristics of the interviewees.

## Abbreviations

BP	Blood pressure
GOPC	General outpatient clinic
HK	Hong Kong
HT	Hypertension
PC	Primary care

## References

[1] Mills K.T., Bundy J.D., Kelly T.N., Reed J.E., Kearney P.M., Reynolds K., Chen J., He J. (2016). Global Disparities of Hypertension Prevalence and Control: A Systematic Analysis of Population-Based Studies From 90 Countries. Circulation.

[2] World Health Organization (2020). Improving Hypertension Control in 3 Million People: Country Experiences of Programme Development and Implementation.

[3] Flint A.C., Conell C., Ren X., Banki N.M., Chan S.L., Rao V.A., Melles R.B., Bhatt D.L. (2019). Effect of systolic and diastolic blood pressure on cardiovascular outcomes. N. Engl. J. Med..

[4] Centre for Health Protection Department of Health (2018). Hong Kong Reference Framework for Hypertension Care for Adults in PC Settings (Patient Version). https://www.fhb.gov.hk/pho/files/e_hypertension_care_patient.pdf.

[5] AHA/ACC (2017). 2017 Guideline for the Prevention, Detection, Evaluation, and Management of High Blood Pressure in Adults A Report of the American College of Cardiology/American Heart Association T. J. Am. Coll. Cardiol..

[6] Lu J., Lu Y., Wang X., Li X., Linderman G.C., Wu C., Cheng X., Mu L., Zhang H., Liu J. (2017). Prevalence, awareness, treatment, and control of hypertension in China: Data from 1·7 million adults in a population-based screening study (China PEACE Million Persons Project). Lancet.

[7] Panoulas V.F., Metsios G.S., Pace A.V., John H., Treharne G.J., Banks M.J., Kitas G.D. (2008). Hypertension in rheumatoid arthritis. Rheumatology.

[8] World Health Organization (2013). A Global Brief on Hypertension: Silent Killer, Global Public Health Crisis.

[9] Petrie J.R., Guzik T.J., Touyz R.M. (2018). Diabetes, hypertension, and cardiovascular disease: Clinical insights and vascular mechanisms. Can. J. Cardiol..

[10] Unger T., Borghi C., Charchar F., Khan N.A., Poulter N.R., Prabhakaran D., Ramirez A., Schlaich M., Stergiou G.S., Tomaszewski M. (2020). 2020 International Society of Hypertension global hypertension practice guidelines. Hypertension.

[11] Weiss R.S. (1995). Learning from Strangers: The Art and Method of Qualitative Interview Studies.

[12] Meyer D.Z., Avery L.M. (2009). Excel as a qualitative data analysis tool. Field Methods.

[13] Khatib R., Schwalm J.D., Yusuf S., Haynes R.B., McKee M., Khan M., Nieuwlaat R. (2014). Patient and healthcare provider barriers to hypertension awareness, treatment and follow up: A systematic review and meta-analysis of qualitative and quantitative studies. PLoS ONE.

[14] Jokisalo E., Kumpusalo E., Enlund H., Takala J. (2001). Patients’ perceived problems with hypertension and attitudes towards medical treatment. J. Hum. Hypertens..

[15] Rajpura J., Nayak R. (2014). Medication adherence in a sample of elderly suffering from hypertension: Evaluating the influence of illness perceptions, treatment beliefs, and illness burden. J. Manag. Care Pharm..

[16] Goldbeck R. (1997). Denial in physical illness. J. Psychosom. Res..

[17] Fleming S., Atherton H., McCartney D., Hodgkinson J., Greenfield S., Hobbs F.D., Mant J., McManus R.J., Thompson M., Ward A. (2015). Self-screening and non-physician screening for hypertension in communities: A systematic review. Am. J. Hypertens..

[18] Yang W.Y., Melgarejo J.D., Thijs L., Zhang Z.Y., Boggia J., Wei F.F., Hansen T.W., Asayama K., Ohkubo T., Jeppesen J. (2019). Association of office and ambulatory blood pressure with mortality and cardiovascular outcomes. JAMA.

[19] Bo Y., Kwok K.O., Chung V.C., Yu C.P., Tsoi K.K., Wong S.Y., Lee E.K. (2020). Short-term reproducibility of ambulatory blood pressure measurements: A systematic review and meta-analysis of 35 observational studies. J. Hypertens..

[20] Kaczorowski J., Chambers L.W., Dolovich L., Paterson J.M., Karwalajtys T., Gierman T., Farrell B., McDonough B., Thabane L., Tu K. (2011). Improving cardiovascular health at population level: 39 community cluster randomised trial of Cardiovascular Health Awareness Program (CHAP). BMJ.

[21] Morris Z.S., Wooding S., Grant J. (2011). The answer is 17 years, what is the question: Understanding time lags in translational research. J. R. Soc. Med..

[22] Guirguis-Blake J.M., Evans C.V., Webber E.M., Coppola E.L., Perdue L.A., Weyrich M.S. (2021). Screening for hypertension in adults: Updated evidence report and systematic review for the US Preventive Services Task Force. JAMA.

[23] Wood S., Greenfield S.M., Haque M.S., Martin U., Gill P.S., Mant J., Mohammed M.A., Heer G., Johal A., Kaur R. (2016). Influence of ethnicity on acceptability of method of blood pressure monitoring: A cross-sectional study in PC. Br. J. Gen. Pract..

[24] Shimbo D., Artinian N.T., Basile J.N., Krakoff L.R., Margolis K.L., Rakotz M.K., Wozniak G. (2020). Self-measured blood pressure monitoring at home: A joint policy statement from the American Heart Association and American Medical Association. Circulation.

