# Supplementary material for: Barriers and Determinants to the Underutilized Hypertension Screening in Primary Care Patients in Hong Kong: A Mixed-Method Study
Source: Int J Environ Res Public Health. 2023 Jan 5;20(2):985. doi: 10.3390/ijerph20020985 (PMC9859345; doi:10.3390/ijerph20020985)
Supplement: Supplementary file 1 [file ijerph-20-00985-s001.zip › ijerph-2130252-supplementary.pdf]

Supplementary File S1: Questionnaire

Questionnaire

Please answer all questions.

Name ： ____________________________
Age: _______________________________
Clinic number: ______________________

SECTION 1: General demographic

1. Sex:
   ☐ Male ☐ Female

2. Education level:
   ☐ No formal education ☐Primary school
   ☐ Secondary school ☐ Post-secondary diploma
   ☐ Bachelor degree ☐ Master degree or higher

3. Employment status:
   ☐ Employed ☐ Unemployed ☐ Retired ☐ Student
   ☐ Others ：______________

4. Marital status:
   ☐ Single ☐ Married ☐ Divorced ☐ Others ：______________

5. Household income:
   ☐ Below $15,000 ☐ $15,001 to $30,000 ☐ $30,001 to $45,000 ☐ $45,001 to $60,000
   ☐ $60,001 to $75,000 ☐ $75,001 to $90,000 ☐ Over $90,001

6. Health insurance status:
   ☐ Covered ☐ Not covered

SECTION 2: Health habit

1. Do you have any diagnosed chronic diseases?
   ☐ Yes ☐ No ☐ Don’t know

2. Tobacco smoking status:
   ☐ Current smoker ☐ Ex-smoker ☐ Never smoker

3. Do you have regular drinking habit?
   ☐ Daily drinker ☐ Social drinker ☐ Ex-drinker ☐ Never drinker

4. Does your family member have hypertension?
☐ Yes ☐ No ☐ Don't know

5. Do you have a regular family doctor?
☐ Yes ☐ No

6. Have you attended to any clinic in the past 2 years?
☐ Yes ☐ No

7. Have you ever measured your blood pressure?
☐ Yes ☐ No ☐ Don't know

8. Have you measured your blood pressure in the past 2 years?
☐ Yes ☐ No ☐ Don't know

Part III: Health data measurement

Blood pressure:
Left arm: __________mmHg
Right arm: ____________mmHg
Further 2 readings from the arm with higher reading
1st reading: _______________mmHg
2nd reading: _______________mmHg

Body weight: ___________kg
Body height; ____________m

9. Do you think your blood pressure reading as normal level?
☐ Yes ☐ No ☐ Don't know

10. Thank you for your response. Your response is very important for us to understand why people check their blood pressure. We may choose 20 participants in the future for person interview. May you give us your contact number so that we may contact you in future?

Tel no,:________________________________

End of questionnaire.
Thank you for your kind participation.

Supplementary File S2 Interview guide

For interviewer:

The purpose of this discussion will be to understand:

1), your concerns and perceptions on hypertension including prognosis and mobility,

2), your perspective of your current blood pressure measurement,

3), your concerns and perceptions on not measuring blood pressure.

Introduction:

This is _____ from the JC School of Public Health and Primary Care, The Chinese University of Hong Kong. Thank you for taking my phone call. You are invited to this phone interview because you aged over 18, not diagnosed hypertension, diabetes mellitus, stroke, heart diseases and chronic kidney diseases before, measured to have an elevated BP and have a reluctance on measuring blood pressure and without physical or mental illness that prevented the BP measurements. I will read the consent form first, and let you know better about the purpose of this interview. After obtaining your consent, I will ask you about 7-8 questions. It may take about 15-30 minutes. Is it okay?

- *If yes, then proceed to read the consent form.*
- *If no, then thank the interviewee and end the phone call.*

Supplementary File S2: Interview guide (cont.)

Main questions and follow-up questions used for interviewing patients

Topics	*Main questions*	*Follow-up questions*	*Probes*
Knowledge questions	*Can you tell me about what you know about your measurement of blood pressure?*<br><br>*Can you tell me about what you know about hypertension?*	*What do you think about it?*<br><br>*What have you heard from others or sources?*	*Anything else about this*<br><br>*Anything else about this*
Sources of information	*Where did you hear about the prognosis of hypertension?*	*What did they say about this?*	*Tell me more about that.*
Experience	*Please tell me about your experience of clinic visit (e.g. treatment, arrangement, cost)?*	*What is your opinion on the overall experience?*	*Anything else you have experienced that want to elaborate more?*
Opinion/ value questions	*In your opinion, do you have concerns on your blood pressure level?*<br><br>*How do you view about the experience of measuring blood pressure?*	*How do you see the blood pressure measurement may or may not benefit to your health?*<br><br>*What influenced your thought about that?*	*Why do you think on that way?*<br><br>*Can you elaborate on this?*

	How do you view about the fact that you are having hypertension?	*What influenced your thought about that?*	*Can you tell me more?*
	What are the barriers hesitate you to measure blood pressure in the past?	*What influenced your thought about that?*	*Why do you think on that way?*
	In your opinion, how can we improve to increase the public awareness on hypertension?	*What influenced your thought about that?*	*Can you elaborate on this?*

Supplementary Table S1. Characteristics of the interviewees

Participant	Age	Gender	Marital Status	Employment	Clinical visit in 2-years	Self-perceived HT condition
1	65	Female	Married	Unemployed	Yes	Normal
2	24	Male	Not married	Employed	No	Normal
3	80	Female	Married	Unemployed	Yes	Abnormal
4	53	Female	Married	Employed	Yes	Normal
5	43	Male	Not married	Employed	Yes	Abnormal
6	19	Male	Not married	Unemployed	No	Abnormal
7	62	Male	Married	Unemployed	Yes	Abnormal
8	58	Female	Married	Unemployed	No	Abnormal
9	70	Male	Married	Unemployed	Yes	Abnormal
10	72	Female	Married	Unemployed	No	Abnormal
11	24	Male	Not married	Employed	No	Abnormal
12	63	Female	Not married	Unemployed	No	Abnormal
13	74	Male	Married	Employed	Yes	Abnormal
14	75	Male	Married	Unemployed	Yes	Abnormal
15	22	Male	Not married	Employed	No	Normal
16	18	Female	Not married	Unemployed	Yes	Normal
17	50	Male	Not married	Unemployed	Yes	Abnormal
18	31	Male	Not married	Employed	Yes	Normal
19	52	Male	Married	Employed	No	Abnormal
20	83	Male	Married	Unemployed	No	Abnormal
21	81	Male	Married	Unemployed	No	Abnormal
22	72	Male	Married	Unemployed	Yes	Abnormal
23	22	Male	Not married	Employed	Yes	Abnormal
24	65	Female	Married	Unemployed	Yes	Normal
